# The Impact of Mitochondrial Dysfunction on Dopaminergic Neurons in the Olfactory Bulb and Odor Detection

**DOI:** 10.1007/s12035-020-01947-w

**Published:** 2020-06-20

**Authors:** Thomas Paß, Marlene Aßfalg, Marianna Tolve, Sandra Blaess, Markus Rothermel, Rudolf J. Wiesner, Konrad M. Ricke

**Affiliations:** 1 Center for Physiology and Pathophysiology, Institute of Vegetative Physiology, University of Cologne, Cologne, Germany; 2 Neurodevelopmental Genetics, Institute of Reconstructive Neurobiology, University of Bonn School of Medicine & University Hospital Bonn, Bonn, Germany; 3 Institute for Biology II, Dept. Chemosensation, AG Neuromodulation, RWTH Aachen University, Aachen, Germany; 4 Cologne Excellence Cluster on Cellular Stress Responses in Aging-associated Diseases (CECAD), University of Cologne, Cologne, Germany; 5 Present Address: Ottawa Hospital Research Institute, Brain and Mind Institute, University of Ottawa, 501 Smyth Rd., Ottawa, ON K1H8M5 Canada

**Keywords:** Parkinson’s disease, Mitochondrial dysfunction, Olfactory bulb, Neurogenesis

## Abstract

Understanding non-motor symptoms of Parkinson’s disease is important in order to unravel the underlying molecular mechanisms of the disease. Olfactory dysfunction is an early stage, non-motor symptom which occurs in 95% of Parkinson’s disease patients. Mitochondrial dysfunction is a key feature in Parkinson’s disease and importantly contributes to the selective loss of dopaminergic neurons the *substantia nigra pars compacta*. The olfactory bulb, the first olfactory processing station, also contains dopaminergic neurons, which modulate odor information and thereby enable odor detection as well as odor discrimination. MitoPark mice are a genetic model for Parkinson’s disease with severe mitochondrial dysfunction, reproducing the differential vulnerability of dopaminergic neurons in the midbrain. These animals were used to investigate the impact of mitochondrial dysfunction on olfactory-related behavior and olfactory bulb dopaminergic neuron survival. Odor detection was severely impaired in MitoPark mice. Interestingly, only the small anaxonic dopaminergic subpopulation, which is continuously replenished by neurogenesis, was moderately reduced in number, much less compared with dopaminergic neurons in the midbrain. As a potential compensatory response, an enhanced mobilization of progenitor cells was found in the subventricular zone. These results reveal a high robustness of dopaminergic neurons located in the olfactory bulb towards mitochondrial impairment, in striking contrast to their midbrain counterparts.

**Electronic supplementary material:**

The online version of this article (10.1007/s12035-020-01947-w) contains supplementary material, which is available to authorized users.

## Introduction

Parkinson’s disease (PD) is the most common movement disorder affecting 1% of the population above 60 years [1] characterized by the initial loss of midbrain dopaminergic neurons (DaNs) in the *substantia nigra pars compacta* (SNc). The depletion of striatal dopamine leads to progressive and irreversible motor impairment [2]. Besides the cardinal symptoms of PD, non-motor dysfunctions including depression, sleep disturbance, and hyposmia have been described. Olfactory dysfunction is found in more than 95% of PD patients and can precede motor symptoms by up to 10 years [3–5].

The olfactory bulb (OB) is the first station of sensory processing in the olfactory system. Besides the midbrain, DaNs are also present in the OB and make up 5% of the neuronal population [6]. They function as interneurons and are necessary for odor discrimination rather than for odor detection [7, 8]. They are primarily located in the glomerular layer (GL), where they modulate the activity of both olfactory sensory fibers [9, 10] and mitral cells by D_2_ receptor-mediated inhibition [11, 12] as well as via contacts with external tufted cells [13]. Despite of multiple as well as contradictory statements regarding their categorization, OB DaNs can be clearly divided into small (5–10 μm), anaxonic (SCs) and large (10–15 μm diameter), axonic cells (LACs) [14]. In contrast to LACs, SCs can be continuously generated throughout life by neurogenesis in mice as well as in humans [15–19]. Progenitor cells are formed in the dorsolateral region of the subventricular zone (SVZ) [20] and are characterized by the expression of the transcription factor PAX6, required for development into the DaN phenotype [21–23]. The majority of progenitor cells in the OB differentiate into interneurons in the granule cell layer, while only 5% of the 20,000–30,000 newborn cells generated daily migrate to the GL [24, 25].

In PD, there are contradictory results according to the fate of DaNs in the OB. *Luquin and colleagues* reported elevated numbers of periglomerular DaNs, potentially displaying a compensatory mechanism induced by the early degeneration of other neurotransmitter systems and resulting in the olfactory dysfunction of patients [26]. In contrast, no difference in OB DaN numbers were found between PD patients and healthy individuals, implying that PD-related hyposmia is not due to alterations in the quantity of OB DaNs [27, 28].

Mitochondrial dysfunction is a central feature of PD, both in the common idiopathic as well as in the rare familiar forms caused by mutations, e.g. in the Parkin, Pink1, LRRK2, or DJ-1 genes. During normal aging, SNc DaNs accumulate high loads of deletions in mitochondrial DNA (mtDNA), present in thousands of copies in neurons. Interestingly, this is accompanied by an upregulation of mtDNA copy numbers in healthy humans, while this compensatory mechanism is disrupted in PD patients [29]. This indicates that a defective mtDNA maintenance system and subsequent severe mitochondrial impairment is an important factor for the degeneration of SNc DaNs in PD.

The mitochondrial transcription factor A (TFAM) is crucial for mtDNA transcription and maintenance [30, 31]. Depletion of TFAM consequently leads to the loss of mtDNA encoded transcripts followed by a respiratory chain defect. MitoPark mice are lacking TFAM exclusively in DaNs, which culminates in progressive neuronal death and corresponding motor impairment starting from 14 weeks of age [32, 33]. The progression of PD is recapitulated in terms of both anatomical and behavioral malfunctions. However, the impact of mitochondrial dysfunction on OB DaNs has not been investigated so far. Therefore, olfactory-related behavior was explored in MitoPark mice. Furthermore, mice were used to study the impact of mitochondrial defects on OB DaN survival as well as adult neurogenesis.

## Materials and Methods

### Experimental Model

All experiments were conducted in agreement with European and German guidelines and approved by local authorities (LANUV NRW; 81–02.04.2018-A210) (for breeding details, see supplementary methods). Experiments were carried out with male or female mice of the strain C57/BL6N. For the generation of MitoPark mice, DAT-*cre* mice (Cre-gene inserted upstream of the translation start codon in exon 2 of the DAT gene) and animals with a loxP-flanked *Tfam* allele were crossed as described in detail previously [32]. MitoPark mice (genotype *Tfam*^*loxP/loxP*^, +/DAT-*cre)* show homozygous disruption of *Tfam* in dopaminergic neurons. *Tfam*^*loxP/loxP*^ and *Dat-cre* mice were provided by Nils-Göran Larsson (Max-Planck-Institute for Biology of Ageing, Köln, Germany). *Tfam*^*loxP/WT*^ or *Tfam*^*loxP/loxP*^ mice were used as controls.

### COX-SDH Histochemistry

Visualization of cytochrome c cxidase (COX) deficiency was performed by COX-SDH enzymatic activity staining [34]. COX is a respiratory chain (RC) complex, which is partially encoded by mtDNA, while succinate dehydrogenase (SDH), another respiratory chain enzyme, is entirely encoded by nuclear DNA. Impaired integrity of mtDNA results in COX-deficiency, but sustained SDH activity. Cells with decreased COX activity will stain blue, while cells with normal COX activity will appear brown (for details, see supplementary methods). Quantification of COX-deficient cells was performed at Bregma + 4.30 mm and + 3.00 mm. Four images (33 × 50 μm) were taken from dorsal, ventral, and lateral OB regions per slice each. Number of COX-deficient cells was defined per slice.

### Immunohistochemistry

Brain sections were stained for tyrosine hydroxylase (TH) to visualize DaNs and their projections (for details, see supplementary methods).

#### Soma size quantification of OB DaN subpopulations

Soma sizes of DaNs stained for TH were analyzed by using FIJI-software (https://imagej.net/Fiji/Downloads). Cells were selected and automatically measured by the Wand-Auto-Tool based on the black-white contrast of the cells compared with background. The outline of the cell soma was detected, and the area within the shape was automatically calculated. Two subpopulations of DaNs with distinct morphological characteristics have been described [35]. DaN somata in the GL were differentiated into areas of 20–80 μm^2^ for SCs and 80–180 μm^2^ for LACs. Two consecutive brain sections per mouse were analyzed at Bregma +4.3, +3.75, and +3.21. Images were taken from both dorsal and ventral OB regions (for details, see supplementary methods).

### Analysis of Immunohistochemical Stainings

Automated bright-field microscopy was done with a slide scanner (SCN400, Leica) equipped with a 40× objective. High-resolution images from the OB and striatum were generated by the Leica SlidePath Gateway and the Microsoft Image Composite Editor software. TH-positive striatal fiber density was determined by optical density (OD) analysis using FIJI-software (area fraction, https://imagej.net/Fiji/Downloads) with two consecutive sections per mouse at Bregma +0.74 mm. Nonspecific background signal of the *corpus callosum* was subtracted from the striatal OD values. Fluorescence images were obtained by utilizing an inverse confocal microscope (TCS SP8 gSTED, Leica) with a 40× oil objective and the Leica Application Suite (LAS) 3 software. OB fluorescence microscopy was performed at Bregma +4.30 mm, +3.30 mm, and +2.30 mm. TH- as well as PAX6-expressing cells were detected independently before merging the channels and quantifying the number of colocalizing cells, respectively. PAX6 expression in the SVZ was performed at Bregma +1.10 mm, +0.70 mm, and +0.30 mm. Sections immunostained for EYFP/TH were imaged using with an inverted Zeiss AxioObserver Z1 microscope equipped with an ApoTome. Fluorescence images were acquired with Zeiss AxioCam MRm 1388 × 1040 pixels (Carl Zeiss). At 10× (EC PlnN 10x/0.3, Carl Zeiss), tile images were acquired with conventional epifluorescence. At 20× (EC PlnN 20x/0.5, Carl Zeiss), tile images were acquired using the ApoTome function.

### Odor Discrimination Test

Odor discrimination ability was examined to analyze DaN functionality in the OB. In addition, the odor discrimination test provides an opportunity to investigate odor detection rather independently from motor activity (for details of the test, see supplementary methods).

### Statistics

Statistical analysis was done with GraphPad Prism 4 for Windows. Quantified data in the figures and in the text is presented as mean + SEM. Relative data is shown as percentage of control experiments. Values of sample size (*n*) refer to mouse numbers. Unpaired *t* tests, one-way or two-way ANOVA with post hoc comparisons (Bonferroni post hoc test) were used to determine differences between groups. A significance level of 0.05 was accepted for all statistical tests. Asterisks mark *P* values of 0.05 (*), 0.01 (**), 0.001 (***), or 0.0001 (****).

## Results

### Time Course of TFAM Loss in Different DaN Populations

**Fig. 1:**
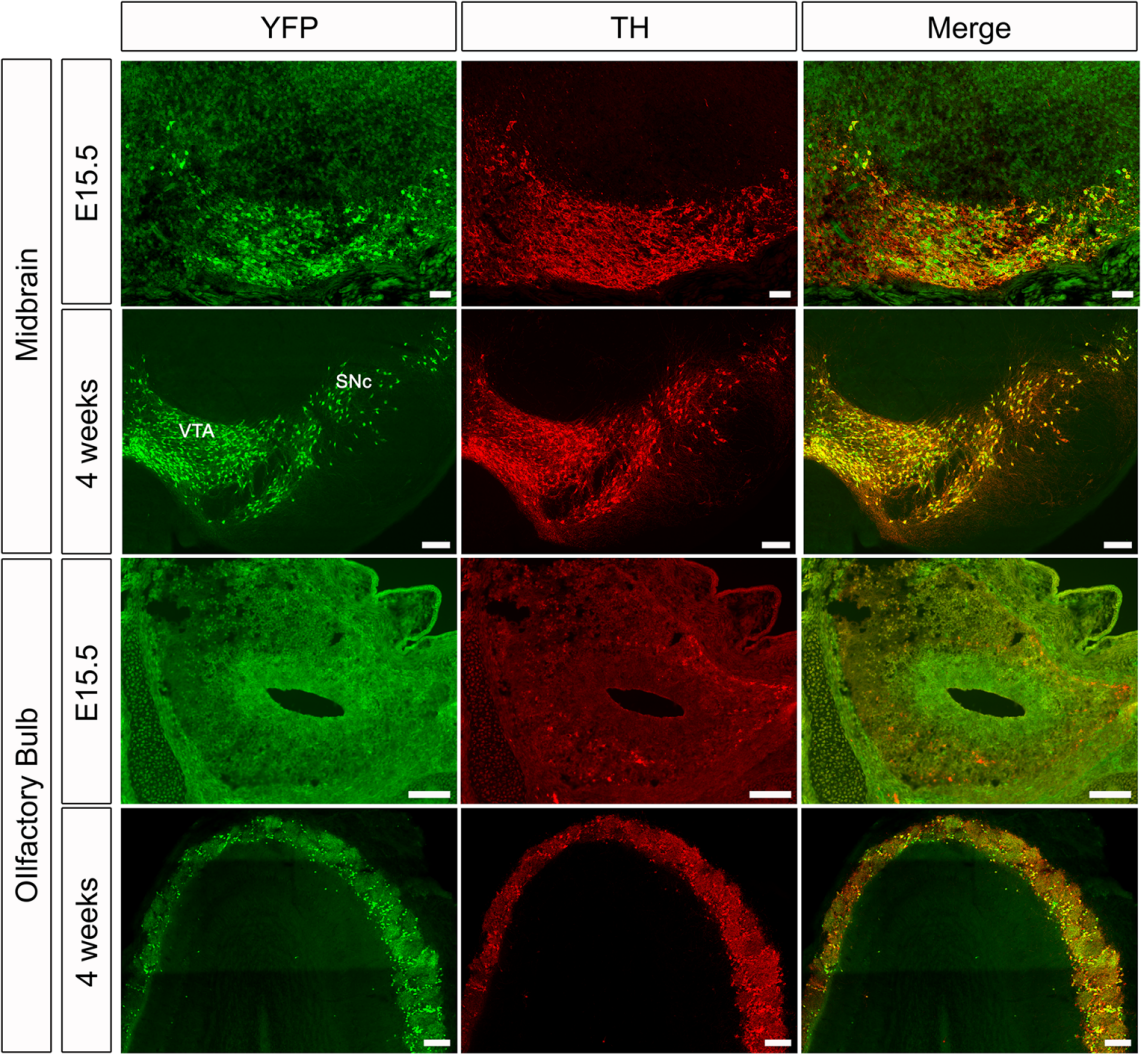
Colocalization of TH and YFP is delayed in OB DaNs. In the midbrain, TH-positive cells are co-expressing YFP at both E15.5 and 4 weeks of age (yellow in merged panel). In contrast, colocalization of TH and YFP in the OB becomes apparent only at the age of 4 weeks. Scale bars: 100 μm

To determine the onset of Cre-mediated recombination, and thus TFAM knockout, in DaNs in the Dat^Cre^ mouse line, we crossed *Dat*^*Cre/+*^ mice with a reporter mouse line expressing enhanced yellow fluorescent protein (EYFP) (*Rosa26*^*loxP-stop-loxP-EYFP*^) [36]. In the midbrain, YFP-expression starts in laterally located TH-positive DaNs at E13.5 and spreads to medial DaN populations by E15.5 (Fig. 1) [37]. In the OB, we could not detect any YFP-expression in TH-positive DaNs at E15.5 (Fig. 1) or P0 (not shown), although some TH-positive neurons were present. Only at 4 weeks of age, TH-positive DaNs expressed YFP, indicating that recombination in OB DaNs only occurs postnatally in *Dat*^*Cre*^ mice. Consistent with this, RNA in situ hybridization data from the Allen Brain Atlas show that *Dat* is not expressed in the OB at P4 [38], while by P14, weak expression of DAT is detected in the OB and this is maintained into adulthood. These data suggest that inactivation of TFAM in the MitoPark model occurs at least 2 weeks later in OB DaNs than in midbrain DaNs.

### MitoPark Mice Show Dopaminergic Nigro-Striatal Degeneration

**Fig. 2:**
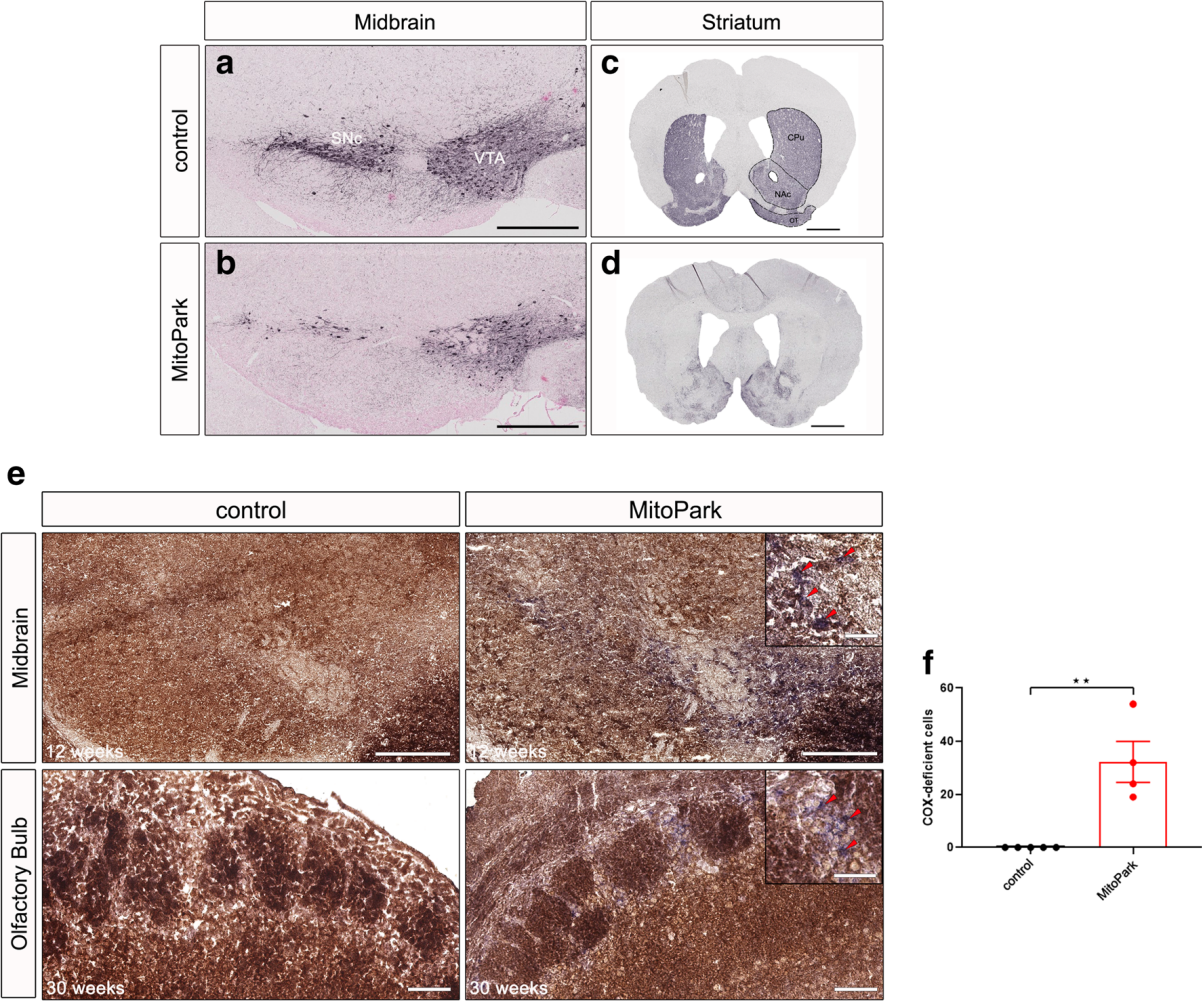
Degeneration of midbrain DaNs and striatal projections in 30-week-old MitoPark mice. **a**, **b** Tyrosine hydroxylase (TH) immunohistochemistry in the midbrain of MitoPark and age-matched control mice showing severe neuronal loss in the *ventral tegmental area* (VTA) and the *substantia nigra pars compacta* (SNc). **c**, **d** Striatal staining presenting the reduction in TH-positive projection area in the *nucleus accumbens* (NAc), *caudate putamen* (CPu), and the *olfactory tubercle* (OT). Three control and four MitoPark mice were analyzed. **e** COX-deficient DaNs in the OB of 30-week-old MitoPark mice. Neurons with reduced activity of cytochrome c oxidase (COX; brown) were unmasked by COX-SDH double staining (blue). In the midbrain, first COX-deficient cells become apparent at 12 weeks of age. Conversely, COX-deficient cells were found in the OB only after 30 weeks. **f** COX-deficient cells in the OB of 30-week-old MitoPark mice. Quantitative analysis revealed a significantly higher number of blue cells in the OB of MitoPark mice when compared with control animals. Control mice: black bar *n* = 5; MitoPark mice: red bar *n* = 4. Scale bars: 500 μm (**a**, **b**), 1 mm (**c**, **d**), midbrain 500 μm, enlarged 50 μm; olfactory bulb 100 μm, enlarged 50 μm (**e**)

TFAM depletion causes progressive motor impairment evoked by the loss of midbrain SNc DaNs and corresponding striatal fibers in the *caudate putamen* (CPu) starting from 14 weeks of age [32, 33]. Since *Tfam* inactivation in OB DaNs occurs later, 20- and 30-week-old MitoPark mice were used in this study. The severe degeneration of the nigro-striatal and the mesolimbic pathway was confirmed in 30-week-old MitoPark mice. In particular, the reduction of TH-positive cells in the SNc and *ventral tegmental area* (VTA) (Fig. 2a, b) as well as TH-staining in the corresponding striatal projection areas is dramatic (Fig. 2c, d). In addition, the *olfactory tubercle* (OT), located in the ventral striatum and innervated by VTA DaNs, shows lowered TH-staining.

### Respiratory Chain Defects in OB DaNs of MitoPark Mice

Loss of TFAM is accompanied by loss of mtDNA-encoded transcripts [32]. Consequently, respiratory chain defects become apparent in midbrain DaNs of 12-week-old MitoPark mice even before the onset of neurodegeneration [39]. In order to verify mitochondrial impairment in OB DaNs, a histochemical staining procedure for COX/SDH activity was performed (Fig. 2e). Sporadically, COX-deficient cells were found in the GL of the OB in MitoPark mice; however, no COX-deficient cells were observed in any of the control animals. These data reveal that COX-deficiency also occurs in OB DaNs of MitoPark mice after inactivating the *Tfam* gene driven by Dat-Cre recombination.

### MitoPark Mice Exhibit Impaired Odor Detection

To assess OB functionality in MitoPark mice, the olfactory behavior of control and MitoPark mice was investigated at different stages. In the buried pellet test, the latency to locate a food pellet, either hidden underneath the bedding or visible on the surface, respectively, was tested (for details of this widely used test, see supplementary methods). There was no significant difference in the latency time to detect the buried food pellet between MitoPark and control mice in any of the investigated ages (Fig. S1a). However, at the age of 30 weeks, MitoPark mice took significantly longer to find the visible pellet (Fig. S1b, control 15.14 ± 5.21 s, MitoPark 134.65 ± 48.14 s, two-way ANOVA, *P* < 0.05), indicating that the severe motor impairment of 30-week-old MitoPark mice may influence the result rather than showing exclusively an affected ability for odor detection.

**Fig. 3:**
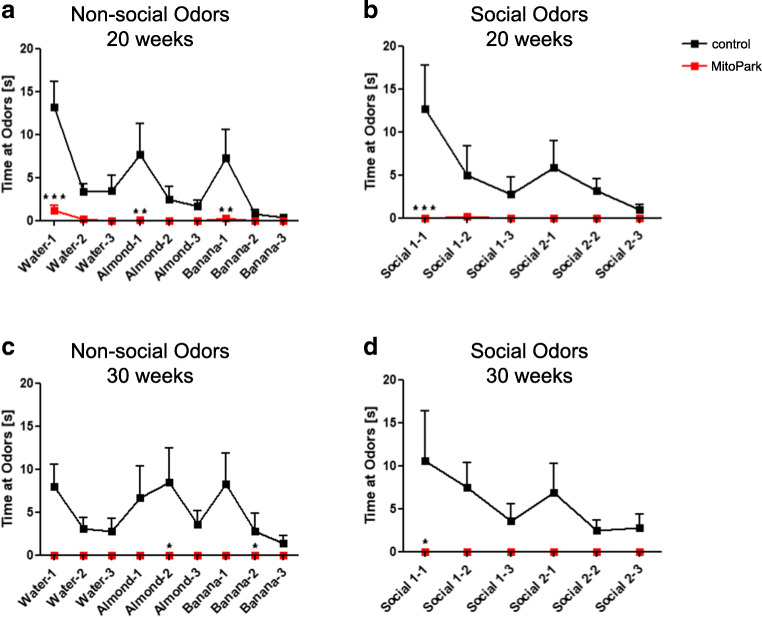
Impaired odor detection in MitoPark mice. **a**–**d** Time spent sniffing at the odor by 20 (**a**, **b**) and 30-week-old (**c**, **d**) MitoPark (red squares) and control mice (black squares). 20 weeks: (*n* = 9–10 mice); 30 weeks: (*n* = 5–6 mice)

In order to analyze odor discrimination as well as odor detection more independent from motor performance, the odor discrimination test was carried out in 20- and 30-week-old MitoPark and control mice. Both non-social and social odors were presented consecutively three times each. Control animals revealed typical olfactory memory and discrimination. They presented habituation behavior to identical odors characterized by decreasing sniffing times as well as dishabituation to new odors with increasing sniffing times (Fig. 3a–d). For MitoPark mice, the time spent at the odors was dramatically reduced and they were not able to detect any of the presented odors. The time spent sniffing at the odor was significantly decreased in 20-week-old MitoPark mice for all odor types (Fig. 3c, d, water-1: two-way ANOVA, *P* < 0.001, almond-1: *P* < 0.01, banana-1: *P* < 0.01, social 1–1: *P* < 0.001). Furthermore, 30-week-old MitoPark mice spent no time at any of the presented odors (Fig. 3c, d). These results indicate that odor detection is fundamentally impaired in MitoPark mice.

### Reduction of Dopaminergic SCs in the OB of MitoPark Mice

**Fig. 4:**
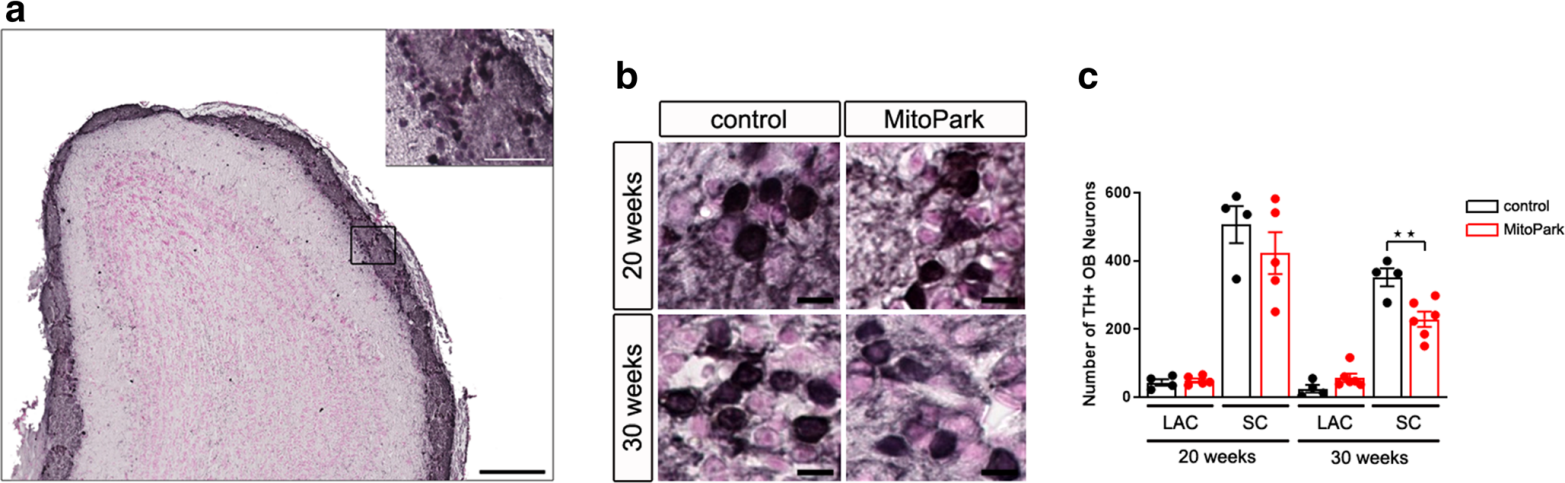
Reduced number of SCs in the OB of MitoPark mice. **a** TH immunohistochemistry of the OB and the enlarged glomerular layer. **b** TH staining in MitoPark and age-matched control mice. **c** Quantification of DaN subpopulations showed a reduction in SCs in the OB of 30-week-old MitoPark mice. In addition, the amount of SCs was higher compared with LACs (20 weeks, control 506.25 ± 54.54 SCs vs. 43.25 ± 9.32 LACs, one-way ANOVA, *P* < 0.0001; MitoPark 422.20 ± 61.70 SCs vs. 49.00 ± 5.81 LACs, *P* < 0.0001; 30 weeks, control 351.25 ± 26.06 SCs vs. 24.75 ± 11.25 LACs, *P* < 0.0001; MitoPark 228.67 ± 22.50 SCs vs. 57.17 ± 12.24 LACs, *P* > 0.01). Control mice: black bars, 20 weeks *n* = 4, 30 weeks *n* = 4; MitoPark mice: red bars, 20 weeks *n* = 5, 30 weeks *n* = 6. Scale bars: 200 μm and 50 μm (**a**), 10 μm (**b**)

Soma size–based quantification was performed to examine the number of DaNs in the two main subpopulations (Fig. 4a, b). The number of SCs was higher compared with LACs in this area, in line with previously reported observations in wt mice [40]. The number of LACs and SCs did not differ between both groups at the age of 20 weeks, however, comparison of control and MitoPark mice showed a decreased number of SCs in 30-week-old MitoPark (Fig. 4c, control: 351.25 ± 26.06, MitoPark: 228.67 ± 22.50, unpaired *t* test, *P* < 0.01). These results suggest that TFAM depletion in OB DaNs preferentially affects SC survival and that this may lead to the observed odor detection impairment. Alternatively, the continuous generation of progenitor cells could be affected and be the reason for the decreased SC number in MitoPark mice.

### Number of PAX6^+^ DaNs Is Not Altered in the OB of MitoPark Mice

**Fig. 5:**
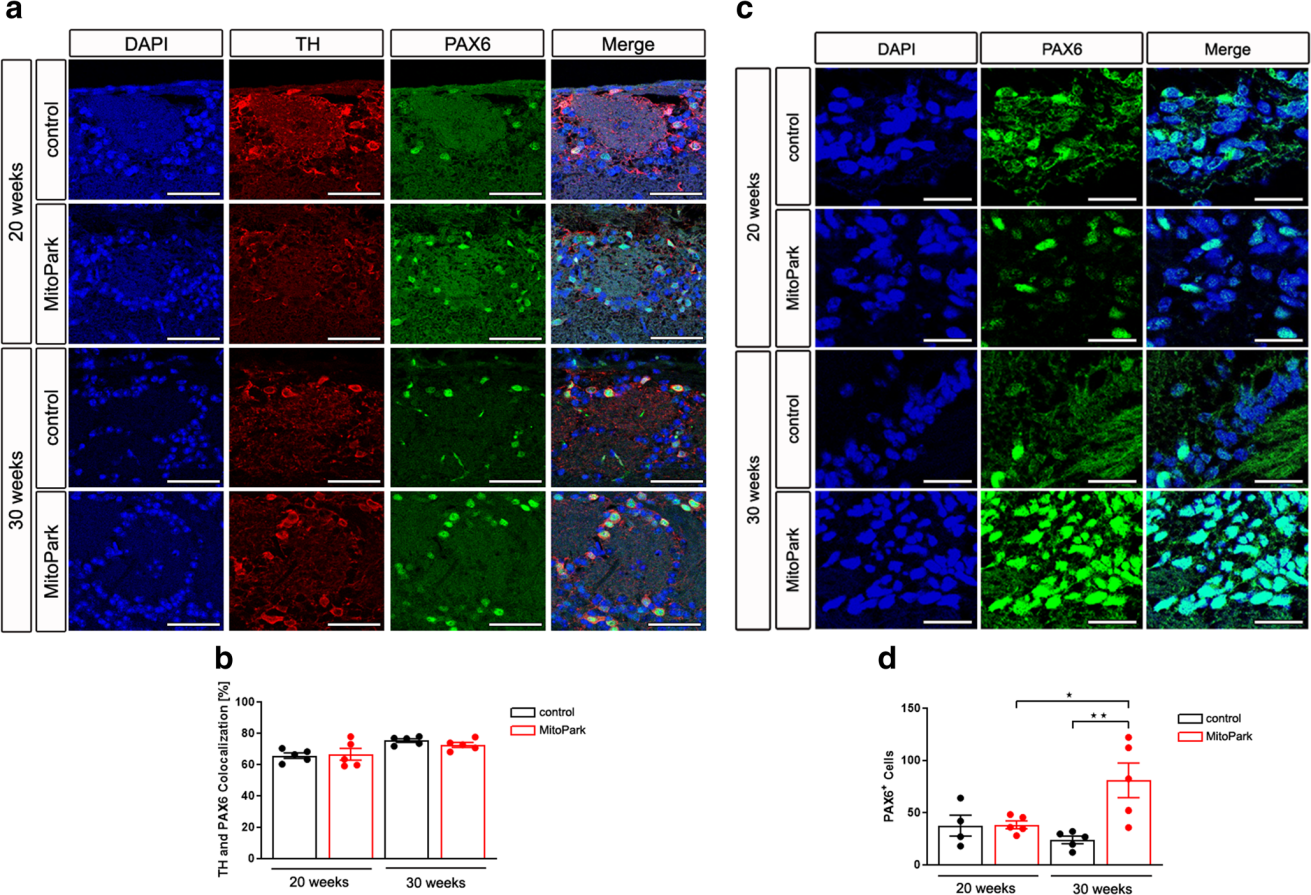
No change in the amount of PAX6-expressing DaNs in the OB but in the amount of progenitor cells in the SVZ of MitoPark mice. **a** Merged TH and PAX6 immunofluorescent staining presents newborn OB DaNs in 20- and 30-week-old MitoPark and age-matched control mice. **b** Quantitative analysis of PAX6-expressing DaN number revealed no difference between MitoPark and control animals. Control mice: black bars, 20 weeks *n* = 5, 30 weeks *n* = 5; MitoPark mice: red bars, 20 weeks *n* = 5, 30 weeks *n* = 5. Increase of PAX6-expressing progenitor cells in the SVZ of MitoPark mice. **c** Combined nuclear (DAPI) and immunofluorescent PAX6 staining depicts the distribution of progenitor cells in MitoPark and age-matched control mice in the SVZ. **d** Quantitative analysis showed an enhanced number of progenitor cells in 30-week-old MitoPark mice. Control mice: black bars, 20 weeks *n* = 4, 30 weeks *n* = 5; MitoPark mice: red bars, 20 weeks *n* = 5, 30 weeks *n* = 5. Scale bars: 50 μm (**a**), 25 μm (**b**)

In contrast to LACs, SCs can be newly generated even postnatally. To investigate if the reduced number of SCs in MitoPark mice is caused by an alteration in progenitor cell differentiation, PAX6 immunohistochemistry was performed in the OB (Fig. 5a). The transcription factor PAX6 is postnatally expressed in progenitor cells of the subventricular zone to mediate their dopaminergic fate [21–23, 41–43] and remains expressed in DaN progenitors after arriving in the OB [44–46]. In addition, PAX6 has recently been used by Höglinger and colleagues as a marker for neurogenic progenitors within the rostral migratory stream, which gives rise to DaNs in the OB [47]. In contrast, PAX6 expression in cells already established during development decreased over time with no colocalization in neurons [48]. This makes PAX6 an ideal marker for adult-born DaNs in the mouse OB. The amount of TH-positive neurons also expressing PAX6 did not differ between MitoPark and control mice (Fig. 5b, 20 weeks: control 65.66 ± 1.79%, MitoPark 66.55 ± 3.76%; 30 weeks; control 75.30 ± 1.17%, MitoPark 72.54 ± 1.59%, one-way ANOVA, n.s.). These data reveal that DaN progenitor cell differentiation in the OB is not impaired in MitoPark mice.

### MitoPark Mice Show Increased Progenitor Cell Mobilization in the SVZ

To further assess whether OB progenitor cell proliferation is affected earlier in the lineage of these neurons, PAX6 expression in the dorsal SVZ was studied (Fig. 5c). At 20 weeks of age, there was no difference between MitoPark and control animals (Fig. 5d, control 37.69 ± 10.00%, MitoPark 38.48 ± 3.71%). However, 30-week-old MitoPark mice revealed a significantly increased number of PAX6-expressing cells when compared with 20-week-old MitoPark mice and age-matched control animals (MitoPark: 20 weeks 38.48 ± 3.71%, 30 weeks 80.80 ± 16.63%, one-way ANOVA, *P* < 0.05; 30 weeks: control 24.02 ± 3.65%, one-way ANOVA, *P* < 0.01). These results indicate an enhanced mobilization of progenitor cells in the dorsal SVZ, probably induced by the decline of SCs in the OB.

## Discussion

In contrast to the midbrain, OB DaNs reveal no Dat-Cre expression during embryonic development (Fig. 1). Moreover, RNA in situ hybridization data [38] suggest that the *Dat* gene only starts to be expressed in OB DaNs in the second postnatal week. Subsequently, cells with respiratory chain deficits become apparent in OB DaNs only in 30-week-old MitoPark mice (Fig. 2e), i.e. 28 weeks after *TFAM* inactivation at around P14, whereas midbrain DaNs uniformly present COX-deficiency already after 12 weeks of age [39], i.e. 13 weeks after *TFAM* inactivation at around E15.5. This reveals a surprisingly different response of different DaN populations to inactivation of mitochondrial gene expression.

To inspect MitoPark mice for OB functionality, olfactory behavior was investigated. The widely used buried pellet test did not reveal olfactory dysfunction in MitoPark mice (Fig. S1A-B), but this may rather be a consequence of the motor impairment at 30 weeks of age. It further has to be noted that the outcome of the buried food pellet test does not exclusively depend on the detection of odors. As a consequence of the bradykinesia, MitoPark mice have to be supplied with moistened food pellets on the surface of the bedding from an age of 15 weeks onward. MitoPark mice are thereby trained to seek for food at the surface by housing conditions. Furthermore, the animals were not food deprived since this would be ethically not permitted with MitoPark mice. In fact, these animals could even suffer from an enhanced desire for food since they start losing weight at 20 weeks of age [49]. Thereby, the buried food pellet test might not be an appropriate test to analyze odor detection impairment in MitoPark mice.

It has further been shown that OB DaNs are involved in olfactory discrimination [7, 50, 51]. In order to analyze discrimination as wells as detection more independently from motor performance and food seeking, the odor discrimination test was performed (Fig. 3a–d). Already 20-week-old MitoPark mice show sharply reduced times spent at all presented odors. After 30 weeks of age, no sniffing time is observed at all, indicating a severe impairment in fundamental odor detection. Noteworthy, the ability of MitoPark mice to move was still sufficient to localize the presented odors (see video sequences in Videos 1 and 2).


Video 1Odor discrimination test of a 30-week-old MitoPark mouse. Movement and sniffing behavior of the test mouse are shown during the presentation of a new odor via a cotton swab in an open cage. While the MitoPark mouse’s ability to move was sufficient to discover the cotton swab, the sniffing behavior appeared undirected. The mouse did not locate the swab at any time in the presented video sequence. (MOV 76648 kb)


Video 2Odor discrimination test of a 30-week-old control mouse. Movement and sniffing behavior of the test mouse are shown while a new odor is presented via a cotton swab in an open cage. The control mouse shows a normal olfactory behavior. In particular, a targeted sniffing behavior is seen, followed by the rapid detection of the odor’s source. (MP4 16818 kb)

In order to investigate the number of DaNs in the GL, soma size–based quantification was performed. Intriguingly, only a reduced number of SCs is found in 30-week-old MitoPark mice, whereas LACs were unaffected (Fig. 4c). In the midbrain of MitoPark mice, both SNc and VTA DaNs present mitochondrial dysfunction at 12 weeks of age, followed by progressive neurodegeneration, with SNc neurons being more affected [32]. This selective vulnerability is one hallmark for PD in patients [52] and raises the question which cell type-specific factors render SNc DaNs vulnerable to mitochondrial dysfunction. Much research has been conducted characterizing neuroanatomical as well as electrophysiological properties of midbrain and OB DaNs. On the one hand, complex axonal morphology might play an essential role regarding the time course of degeneration. SNc DaNs show an extremely large arborization with an estimated number of 100.000–250.000 synapses per neuron [53] compared with both VTA and OB DaNs, with the SCs even being anaxonic [14]. The extended branching results in an extreme bioenergetic demand, leaving SNc DaNs working on a tight energy budget [54], especially when facing additional stressors, such as mitochondrial dysfunction. On the other hand, all three DaN populations are characterized as autonomous pacemakers [40, 55, 56]. The pacemaking machinery of VTA and OB DaNs is mainly driven by a persistent sodium current [40, 57, 58]. Conversely, SNc pacemaker activity is associated with Ca^2+^ influx through plasma membrane Ca_v_1.3 channels, postulated to cause oxidant stress in the mitochondrial compartment [59, 60]. Combined with a low intrinsic calcium buffering capacity, mitochondrial dysfunction leads to an oxidized RedOx-system and hyperpolarized membrane potential in mitochondria of SNc DaNs, which thereby causes neuron death [39].

SCs and LACs differ in various aspects, including morphology, functionality, and neurogenic potential. SCs are anaxonic and thereby generate somatic action potentials with a low firing rate. In contrast, the wide-branching LACs do have an axon and a high firing frequency [19]. SCs are typically type 1 periglomerular cells [61–65]. They receive olfactory nerve and dendrodendritic synapses, which, in turn, lead to the inhibition of mitral cells [66], the principal output neurons of the OB. A reduced number or functional changes of SCs in MitoPark mice could thereby cause temporal shifts in mitral cell activity, previously shown to impair olfactory-related behaviors [67].

In addition, anaxonic SCs are even continuously formed via adult neurogenesis, whereas LACs are exclusively established during embryonic development [15–19]. Consequently, affected neuronal replenishment of SCs could be the explanation for the reduced number of SCs. OB progenitor cells are created in the SVZ of the lateral ventricles and tangentially migrate along the rostral migratory stream before they enter the OB [68]. Those progenitor cells are characterized by the expression of PAX6. Interestingly, MitoPark mice reveal an increased mobilization of PAX6-expressing cells in the SVZ at 30 weeks of age (Fig. 5c, d), indicating a potential compensatory upregulation of progenitor cells. However, the amount of PAX6-expressing DaNs in the OB is stable (Fig. 5a, b). This gives reason to suppose that either the death rate of SCs is so high that the number of replenished neurons in the OB cannot compensate for this or that the upregulation of progenitor cells in the SVZ is not a direct cause of the reduced SC number. In addition, even though the time line of RMS migration is very well established, there is no evidence to when DAT expression is initiated in OB progenitor cells. Potentially, new but not fully matured neurons are more vulnerable to the TFAM knockout due to early DAT expression and die even before reaching the OB.

Besides the reduced number of SCs, it is likely that the severe olfactory dysfunction is caused by other factors. So far no direct link between the midbrain and the OB could be demonstrated [69, 70]. However, a recent study discovered the existence of axonal projections from SNc DaNs to the OB and the ablation of this connectivity resulted in impaired olfactory perception [71]. At 30 weeks of age, MitoPark mice reveal a severe degeneration of SNc DaNs (Fig. 2b). Therefore, the absence of these nigro-bulbar connections could contribute to the olfactory dysfunction in MitoPark mice. More precisely, the loss of SNc-OB projections might lead to Ca^2+^-induced hyperactivity of mitral cells caused by the missing dopaminergic inhibition, as recently demonstrated in a 6-OHDA induced PD mouse model [72]. However, Zhang et al. did not show any data concerning the dopaminergic projection area in the striatum after partial depletion of SNc DaNs, though striatal denervation is likewise affecting olfactory behavior [73]. Since MitoPark mice reveal the degeneration of SNc DaNs as well as corresponding striatal fibers, we postulate that, beside the decreased number of SCs, olfactory dysfunction might be caused by the degeneration of the complete nigro-striatal system. Moreover, the loss of the nigro-striatal system might be the reason for the increased mobilization of progenitor cells in the SVZ observed in 30-week-old MitoPark mice, as shown likewise after 6-OHDA lesioning [74]. Noteworthy, DaNs from the VTA innervate the SVZ and are involved in proliferation of progenitor cells [75]. Increased progenitor cell mobilization may thereby also have its reason in a compensatory upregulation due to the loss of VTA DaNs. More importantly, the SNc and the VTA as well as the striatum further possess dopaminergic projections to higher olfactory brain regions, such as the piriform cortex and the olfactory tubercle [76–78]. Therefore, a prospective disconnection between the nigro-striatal system as well as the VTA and higher olfactory centers might importantly contribute to the impaired olfactory-related behavior in MitoPark mice.

Apart from the dopaminergic system, serotonin could also play a role in the olfactory dysfunction of MitoPark mice. Serotonergic neurons from the raphe nuclei are innervating all layers of the OB [79–81] and deafferentiation of corresponding fibers causes anosmia and OB atrophy [82]. Furthermore, mice that are deficient for *PTEN-induced kinase 1* (*PINK1*), mutations of which make up 1–2% of the familiar forms of PD, possess a decreased serotonergic innervation in the GL, leading to an impaired olfactory behavior [83]. Besides the SNc, the raphe nuclei are also affected in PD patients, with a loss of serotonergic neurons and its projections [84, 85]. However, altered serotonin transporter activities in PD patients failed to correlate with olfactory dysfunction [86], leaving the role of serotonergic neurons in PD-related anosmia unclear.

Our data provide new insights into olfactory dysfunction and adaptations of adult neurogenesis in response to genetic depletion of the dopaminergic system. Furthermore, we show that dopaminergic neurons located in the olfactory bulb reveal a high robustness towards mitochondrial impairment, in striking contrast to their midbrain counterparts.

## Electronic Supplementary Material

Fig. S1 No significant difference in buried food pellet detection between MitoPark and control mice. A) Latency time of MitoPark (red bars) and age-matched control mice (black bars) to the buried and B) unburied food pellet. Control mice: n = 6–23; MitoPark mice: n = 5–18. (PNG 141 kb)

High resolution image (EPS 3127 kb)

ESM 4 (DOCX 48.0 KB)
