# Supplementary figures and images for: The Impact of Mitochondrial Dysfunction on Dopaminergic Neurons in the Olfactory Bulb and Odor Detection
Source: Mol Neurobiol. 2020 Jun 20;57(9):3646–57. doi: 10.1007/s12035-020-01947-w (PMC7398899; doi:10.1007/s12035-020-01947-w)

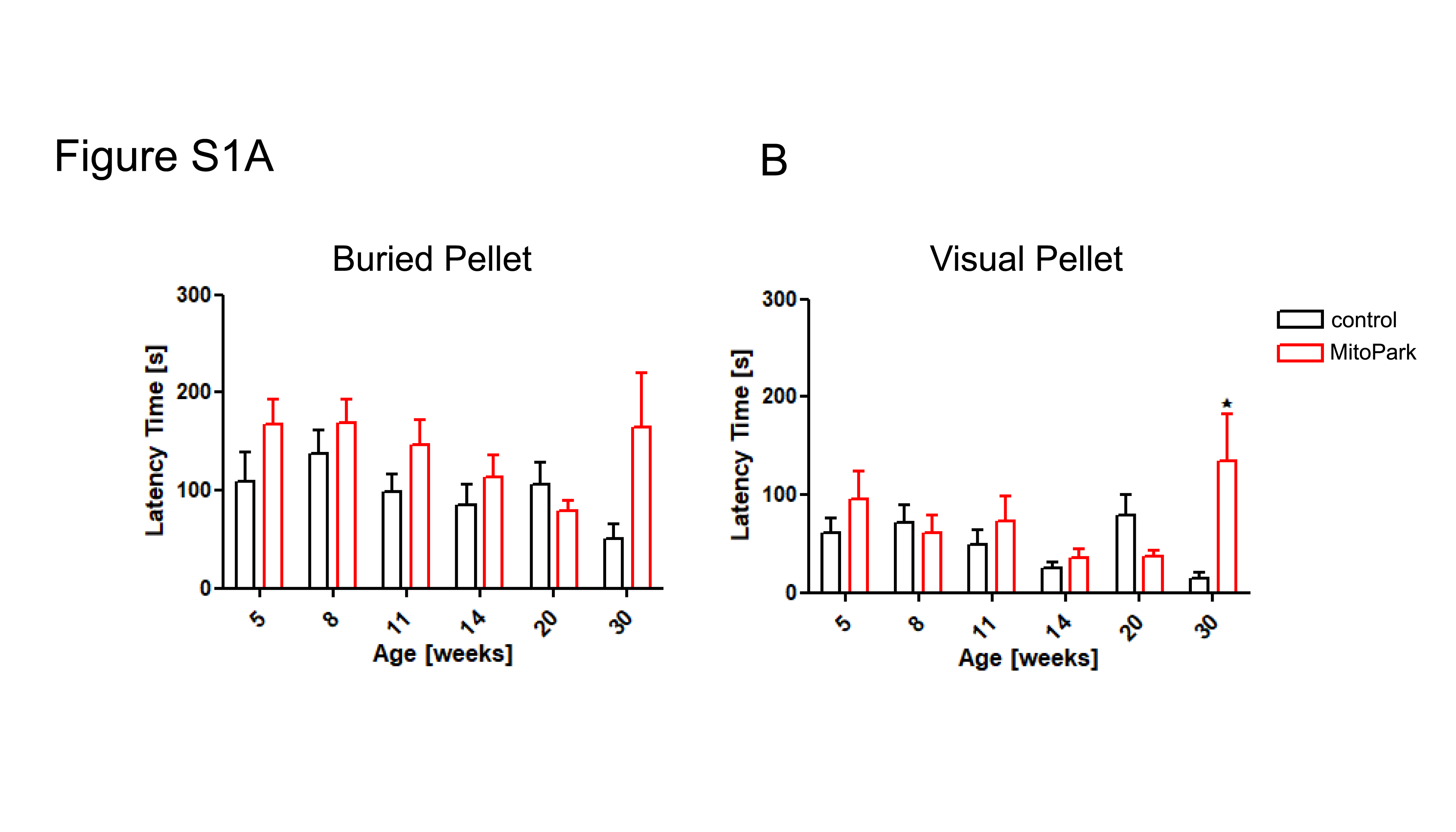

Supplement: Supplementary file 3 — No significant difference in buried food pellet detection between MitoPark and control mice. A) Latency time of MitoPark (red bars) and age-matched control mice (black bars) to the buried and B) unburied food pellet. Control mice: n = 6–23; MitoPark mice: n = 5–18. (PNG 141 kb) [file 12035_2020_1947_Fig6_ESM.png]
