# Supplementary material for: The Impact of Mitochondrial Dysfunction on Dopaminergic Neurons in the Olfactory Bulb and Odor Detection
Source: Mol Neurobiol. 2020 Jun 20;57(9):3646–57. doi: 10.1007/s12035-020-01947-w (PMC7398899; doi:10.1007/s12035-020-01947-w)
Supplement: Supplementary file 5 — (DOCX 48.0 KB) [file 12035_2020_1947_MOESM4_ESM.docx]

### Supplementary methods

### Experimental model

C57/BL6N mice of both sexes were used. *Tfam^loxP/loxP^* and *Dat^Cre^* mice were provided by Nils-Göran Larsson (Köln, Germany).^29^ Both *Tfam^loxP/WT^* and *Tfam^loxP/loxP^* mice were used as controls. Genotypes were identified by PCR using genomic DNA. To analyze the timing and extent of recombination, *Dat^Cre^* mice were crossed with *ROSA^loxP-STOP-loxP-EYFP^* mice*.*^31^

### Genotyping

All genetic lines were identified by qualitative PCR-approaches using genomic DNA either from tail tip or ear punch biopsies. Tissue lysis was done in 75 µL lysis buffer (10 mM NaOH, 0.2 mM EDTA) at 96 °C for 45 min. After adding 75 µL of neutralization buffer (40 mM Tris-HCl, pH 7.6), lysates were centrifuged (1 min, 3000 rpm) and stored at -20 °C. Cycling conditions and primer sequences for the genetic lines are shown in Supplementary Suppl.-Table 1 to Suppl.-Table 4. Agarose gels containing 1.2 or 1.5% agarose (Tfam PCR: 1.5%, DatCre and mito-roGFP PCR: 1.2%) and 0.8 µg/mL ethidium bromide in TAE buffer (40 mM Tris base, 20 mM Acetic acid, 1mM EDTA, pH 8) were used to illustrate PCR products under UV-light.

Suppl.-Table 1: Cycling conditions for Tfam PCR

| cycles | temperature | time |
| --- | --- | --- |
| 1 | 94°C | 120 s |
| 29 | 94°C | 30 s |
|  | 55°C | 45 s |
|  | 72°C | 60 s |

Suppl.-Table 2: Cycling conditions for DatCre PCR

| cycles | temperature | time |
| --- | --- | --- |
| 1 | 94°C | 180 s |
| 35 | 94°C | 30 s |
|  | 61°C | 30 s |
|  | 72°C | 45 s |

Suppl.-Table 3: Genotyping PCR primer sequences

| primer | sequence | T_m_ (elongation temperature) |
| --- | --- | --- |
| Tfam For | 5'CTGCCTTCCTCTAGCCCGGG | 71.1 °C |
| Tfam Rev 1 | 5'GTAACAGCAGACAACTTGTG | 55.8 °C |
| Tfam Rev 2 | 5'CTCTGAAGCACATGGTCAAT | 60.3 °C |
| Dat-Cre For | 5'CATGGAATTTCAGGTGCTTGG | 66.4 °C |
| Dat-Cre Rev 1 | 5'CATGAGGGTGGAGTTGGTCAG | 66.9 °C |
| Dat-Cre Rev 2 | 5'CGCGAACATCTTCAGGTTCT | 64.3 °C |

### **COX-SDH histochemistry**

Following dissection after cervical dislocation, brains were embedded (O.C.T. Tissue-Tek), frozen on dry ice and stored at -80 °C. Brains were cut in 7 µm sections using a cryostat (Leica CM3050 S) and sections were stored at -80 °C. Coronal OB cryosections at Bregma +3.20 mm and midbrain cryosections at Bregma -3.08 mm were air dried and treated with COX incubation solution (100 µM Cytochrome c, reduced; 4 mM diaminobenzidine, 4400 U catalase in 0.1 M phosphate buffer) for 40 min at 37 °C. Afterwards, sections were washed in ddH_2_O and treated with SDH incubation solution (1.5 mM Nitroblue tetrazolium, 130 mM sodium succinate, 200 µM phenazine methosulfate, 1 mM sodium azide) for 180 min at 37 °C. Sections were washed again, dehydrated in 95% and 100% ethanol, air dried and mounted with glycerol-gelatine.

### Immunohistochemistry

Brain sections were stained for tyrosine hydroxylase to visualize DaNs and their projections (details, see supplementary methods). Anaesthetized mice (ketamine/xylazine: 100/10 mg/kg body weight, intraperitoneally) were intracardially perfused consecutively with PBS (GIBCO; 140 mM NaCl, 10 mM sodium phosphate, 2.7 mM KCl, pH 7.4) for 3 min and PBS with 4% para-formaldehyde in PBS for 15 min. Brains were dissected and immersion-fixed in this solution overnight. Afterwards tissues were dehydrated in a series of ethanol solutions (Leica ASP300, Leica Park Tissue Infiltration Machine, CMMC Cologne) and embedded in paraffin (Leica EG1150 H, CMMC Cologne). Coronal 5 µm OB as well as striatal sections were cut with a microtome (Leica RM2125 RTS). Sections were deparaffinized in xylene, washed in a series of ethanol solutions and finally washed in ddH_2_O. For epitope retrieval, sections were heated in citrate buffer (10 mM citric acid monohydrate, pH 6) using a microwave oven.

*Brightfield microscopy*: OB, midbrain and striatal sections were washed in TBS (10 mM Tris base, 150 mM NaCl, pH 7.6, 3 x 5 min), quenched with 0.3% H_2_O_2_/TBS solution and washed again in TBS. Subsequently, sections were blocked in 10% normal goat serum in TBS and afterwards incubated with the tyrosine hydroxylase (TH) antibody (polyclonal rabbit, Abcam, #ab112, 1:750 in 3% skim milk powder/TBS, overnight, 4°C). After another TBS washing step, sections were incubated with the secondary biotinylated antibody (donkey anti-rabbit, dianova, #111-065-006, 1:500 in 3% skim milk powder/TBS, 30 min, RT), followed by blocking with avidin/biotin (Vectastain Elite ABC HRP Kit, Vector Laboratories). Visualization was performed using DAB solution (0.46 mM 3,3’-Diaminobenzidine tetrahydrochloride, 7.3 mM Imidazole, 15.2 mM Ammonium nickel (II) sulfate hexahydrate, 0.015% H_2_O_2_ in TBS, pH 7.1). For OB and midbrain sections, additional counterstaining was performed by using nuclear fast red (NFR, 0.1% in 5% aluminum dissolved 1:5 in ddH_2_O). Following a short washing in ddH_2_O, sections were dehydrated in 50%, 70%, 95% and 100% ethanol, cleared in xylene and mounted with Entellan (Merck Millipore).

**Immunofluorescent staining**

OB and striatal sections were stained for tyrosine hydroxylase, EYFP and PAX6 (preparation and staining details, see supplementary methods). OB and striatal sections rinsed in 0.2% Triton-X 100 in TBS (TBST) after the epitope retrieval and incubated with primary antibodies (TH polyclonal rabbit, Abcam, ab112, 1:750; PAX6 monoclonal mouse, Merck Millipore, MAB5552, 1:500; diluted in Dako antibody diluent, overnight, 4°C). Sections were washed in TBST and incubated with fluorochrome-conjugated secondary antibodies (Goat anti-rabbit TRITC-conjugated, AffiniPure, #111-025-144, 1:1000, Goat anti-mouse DyLight488-conjugated, Jackson-ImmunoResearch, #115-485-003, 1:400). Following another washing step in TBST, midbrain sections were counterstained with DAPI (1 µg/mL in ddH_2_O), washed again in TBST and mounted with Fluoromount (Thermo Fisher Scientific).

Immunofluorescent staining for tyrosine hydroxylase (TH) and EYFP (enhanced yellow fluorescent protein): Pregnant dams were sacrificed by cervical dislocation. Embryos were dissected in ice cold PBS. Heads (embryonic day 15.5) or brains (postnatal day 0) were fixed in 4% paraformaldehyde (PFA) for 120 – 180 min at room temperature (RT). Adult mice were anesthetized and perfused as described above. Tissue was cryopreserved in OCT Tissue Tek (Sakura), embryonic tissue was cryosectioned at 14 μm, adult brains were cryosectioned at 40 μm thickness. Sections were fixed briefly in 4% PFA (5 min at RT), followed by 60 min incubation in 10% NDS in 0.1% Triton in PBS (0.1% PBT). Sections were incubated with primary antibody against EYFP (rat anti-GFP, RRID:AB_10013361, Nalacai, 1:2000) and TH (rabbit anti-TH, RRID:AB_390204, Merck, 1:500) at 4 ̊C in 3% NDS in 0.1% PBT. Sections were washed 3X in 0.1%-PBT and incubated in secondary antibody (donkey anti-rat Alexa 488, RRID:AB_2535794, Thermo Fisher and donkey anti-rabbit Cy3, RRID:AB_2307443, Jackson Immunoresearch) in 3% NDS in 0.1% PBT before mounting with Aqua Polymount (Polysciences Inc.).

### Buried food-pellet test

The buried food-pellet test was carried out in order to assess odor detection capability. Before the test, mice were kept in a new cage (GM500 IVC Green Line, 39.1 cm x 19.9 cm x 16.0 cm) in the experimental room for 45 min to habituate to test conditions. To avoid sensory intraspecific interactions and odor-related distractions, respectively, mice were monitored separately. The 200 mg food pellet (Kellogg’s Frosties®, Kellog GmbH, Germany) was placed approximately 0.5 cm underneath the bedding surface of a second new cage. The pellet’s position was changed at random. The latency to locate the food pellet was defined as the time between the first contact of the mouse with the bedding and the pellet’s discovery. Furthermore, the latency to dig up, characterized by the pellet becoming visible, and to eat the food pellet, defined as being grasped by the mouse in its forepaws, were measured. Conclusively, mice were placed in a third new cage containing a food pellet at the surface of the bedding. The latency to locate the visible pellet was measured analogously to investigate the impact of motor impairment on odor detection.

**Odor discrimination test**

Odor discrimination ability was examined to analyze DaN functionality in the OB. In addition, the odor discrimination test provides an opportunity to investigate odor detection rather independently from motor activity. Mice were kept in a new cage (Type II polycarbonate open cage, 36.5 cm x 20.7 cm x 14.0 cm) in the experimental room for 45 min to habituate to test conditions. During the habituation phase, social odors were collected from housing cages of unfamiliar mice of the same sex. Housing cages were not cleaned for at least three days. Before collecting in a sealable glass jar, cotton swabs were wiped in zigzag fashion across the bottom of the cage and shook off from excess bedding. For non-social odors, water (ddH_2_O), almond and banana extract (AlphaPower Food® Stevia Sweetener, 1:100 in ddH_2_O) were used. Non-social odors (50 µL) were placed freshly on clean cotton swabs immediately before testing. The odor containing swabs were positioned through the water bottle opening by lifting the cage’s wire top and pushing the wooden end of the swab from the underside of the wire top. Swabs were stabilized by laboratory tape. Each odor was presented three times in a row for two minutes with a one-minute break in between. The time the mouse spent sniffing at the odor was measured. Aimed sniffing was defined by direct nose contact with the cotton tip or nose orientation towards the cotton tip approximately 2 cm or closer.
